# Edar is a downstream target of beta-catenin and drives collagen accumulation in the mouse prostate

**DOI:** 10.1242/bio.037945

**Published:** 2019-02-11

**Authors:** Kyle A. Wegner, Vatsal Mehta, Jeanette A. Johansson, Brett R. Mueller, Kimberly P. Keil, Lisa L. Abler, Paul C. Marker, M. Mark Taketo, Denis J. Headon, Chad M. Vezina

**Affiliations:** 1 Molecular and Environmental Toxicology Center University of Wisconsin-Madison, Madison, WI 53706, USA; 2 Department of Comparative Biosciences, University of Wisconsin-Madison, Madison, WI 53706, USA; 3 The Roslin Institute and Royal (Dick) School of Veterinary Studies, University of Edinburgh, Edinburgh EH25 9RG, United Kingdom; 4 MRC Human Genetics Unit, MRC Institute of Genetics and Molecular Medicine, University of Edinburgh, Edinburgh, EH4 2XR, United Kingdom; 5 School of Pharmacy, University of Wisconsin-Madison, Madison, WI 53706, USA; 6 Division of Experimental Therapeutics, Graduate School of Medicine, Kyoto University Yoshida-Konoé-cho, Sakyo, Kyoto 606-8501, Japan

**Keywords:** Prostate, Urogenital sinus, CTNNB1, WNT10B, EDAR, Collagen

## Abstract

Beta-catenin (CTNNB1) directs ectodermal appendage spacing by activating ectodysplasin A receptor (EDAR) transcription, but whether CTNNB1 acts by a similar mechanism in the prostate, an endoderm-derived tissue, is unclear. Here we examined the expression, function, and CTNNB1 dependence of the EDAR pathway during prostate development. *In situ* hybridization studies reveal EDAR pathway components including *Wnt10b* in the developing prostate and localize these factors to prostatic bud epithelium where CTNNB1 target genes are co-expressed. We used a genetic approach to ectopically activate CTNNB1 in developing mouse prostate and observed focal increases in *Edar* and *Wnt10b* mRNAs. We also used a genetic approach to test the prostatic consequences of activating or inhibiting *Edar* expression. *Edar* overexpression does not visibly alter prostatic bud formation or branching morphogenesis, and *Edar* expression is not necessary for either of these events. However, *Edar* overexpression is associated with an abnormally thick and collagen-rich stroma in adult mouse prostates. These results support CTNNB1 as a transcriptional activator of *Edar* and *Wnt10b* in the developing prostate and demonstrate *Edar* is not only important for ectodermal appendage patterning but also influences collagen organization in adult prostates.

This article has an associated First Person interview with the first author of the paper.

## Abstract (precis)

**Summary:** This study provides a rare connection between beta catenin and ectodysplasin A receptor in an endoderm derived tissue and presents a potential mechanism for collagen accumulation in the prostate.

## INTRODUCTION

The mouse prostate derives from the urogenital sinus (UGS), a fetal structure at the base of the bladder consisting of endoderm-derived epithelium, mesoderm-derived mesenchyme and other cell types. Prostate development is initiated by androgen-induced signals from UGS mesenchyme (Cunha and Lung, 1978; Goldstein and Wilson, 1975; Lasnitzki and Mizuno, 1980). Prostate ductal progenitors (prostatic buds) arise as solid epithelial projections in a periodic pattern to establish position and number of mature prostatic ducts.

The Wingless/beta-catenin (Wnt/CTNNB1) is an androgen sensitive signaling pathway critical for prostatic bud formation (He et al., 2018). CTNNB1 and its target genes are present in prostatic buds from the earliest stage of prostate development and continuing at least through postnatal branching morphogenesis (Francis et al., 2013; Simons et al., 2012). Chemical inhibition or genetic deletion of CTNNB1 in UGS epithelium completely prevents prostatic bud formation (Mehta et al., 2013; Simons et al., 2012), while excessive CTNNB1 activation by genetic gain-of-function increases the inter-bud interval and reduces the quantity of prostatic buds formed (Mehta et al., 2013). How CTNNB1 is activated during prostate development and how its activity is restricted to prostatic bud tips is not fully understood.

Ectodysplasin A receptor (*Edar),* a potential CTNNB1 regulatory signaling molecule, is selectively expressed in prostatic bud tips (Keil et al., 2012a, b). EDAR is an essential patterning molecule in ectodermal appendages including primary hair follicles, feathers, mammary glands, salivary glands and teeth (Drew et al., 2007; Jaskoll et al., 2003; Lindfors et al., 2013; Mou et al., 2006; Tucker et al., 2000; Zhang et al., 2009). In these organs, ectodysplasin A (EDA) activates membrane-bound EDAR, which drives NF-kappa B (NF-κB) activation (Mikkola, 2009; Schmidt-Ullrich et al., 2001). NF-κB-dependent synthesis of *Wnt10b* and other target genes maintains CTNNB1 activity in ectodermal placodes and restricts the CTNNB1 activation domain size (Zhang et al., 2009). WNT10B is the earliest known secreted protein expressed by prostate epithelium, it can drive prostatic fate determination in cells and has been recently been identified as a marker of prostate epithelial progenitors (He et al., 2018; Hu et al., 2017).

Co-localization of CTNNB1 target genes with *Edar* and *Wnt10b* in prostatic bud tips (Keil et al., 2012a; Mehta et al., 2011) led us to hypothesize that EDAR is induced by CTNNB1 in the developing prostate and patterns prostatic buds by controlling CTNNB1 domain size as it does in ectoderm-derived appendages. Here, we reveal that *Eda*, *Edar* and *Wnt10b* are expressed during prostatic bud formation, elongation and branching morphogenesis. We demonstrate that ectopic CTNNB1 expression induces *Edar* and *Wnt10b* in UGS epithelium. We use genetic approaches to show that *Edar* is expendable for *in vivo* prostatic bud patterning. Genetically increasing or decreasing expression of *Edar* has no discernable influence on prostatic bud development. However, genetic gain-of-function experiments demonstrate that EDAR overexpression affects prostate stromal composition by resulting in an abnormally thickened fibromuscular stroma containing excessive collagen. This is the first study to demonstrate EDAR and CTNNB1 signaling pathways intersect during formation of an endoderm-derived tissue (prostate) and EDAR activity influences prostatic extracellular matrix organization.

## RESULTS

**Fig. 1.**
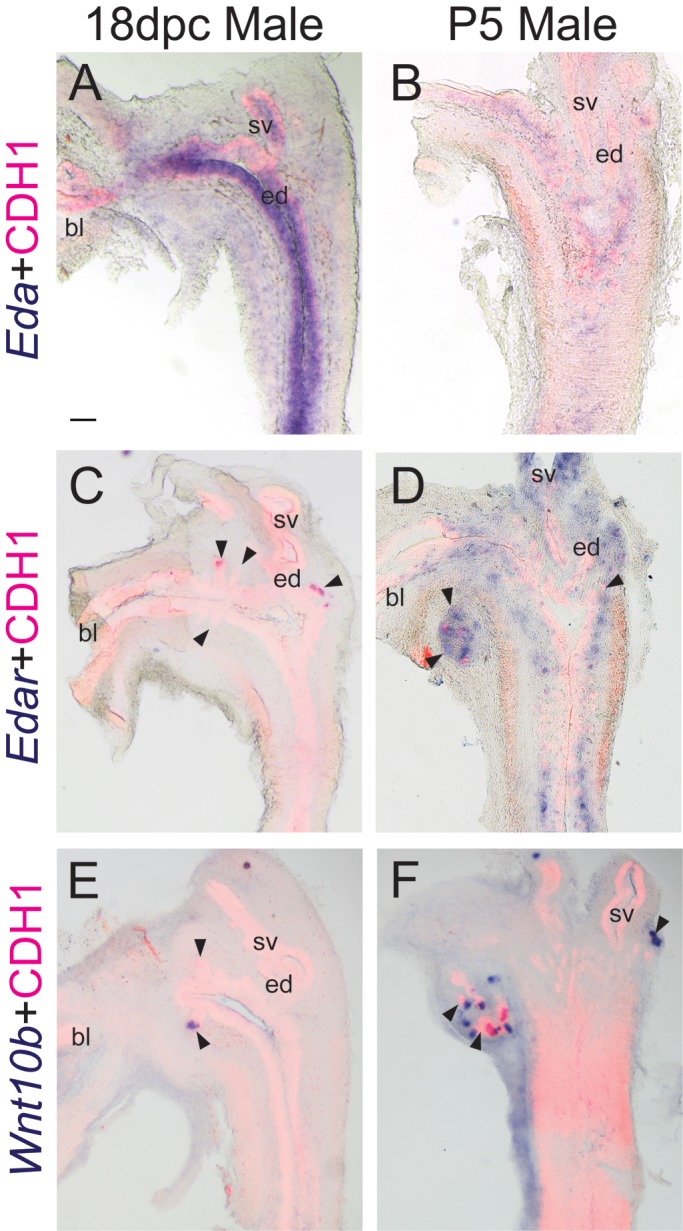
**EDAR signaling pathway mRNA expression patterns in developing and neonatal prostate.** Near mid-sagittal sections (50 µm) of 18 dpc and P5 male LUT were stained by ISH to visualize mRNA expression (purple) patterns of (A,B) *Eda* (C,D) *Edar*, (E,F) *Wnt10b*. Sections were then stained by immunofluorescence with an anti-cadherin 1 (CDH1) antibody that recognizes all epithelium (red). Results in each panel are representative of three males. Arrowheads indicate prostatic buds. bl, bladder; ed, ejaculatory duct; sv, seminal vesicle. All images are of the same magnification. Scale bar: 100 µm.

*Edar* mRNA localizes to prostatic epithelium during bud formation and branching morphogenesis (Keil et al., 2012a). To determine whether other pathway components are expressed, *in situ* hybridization (ISH) was used to visualize the EDAR ligand *Eda* and the putative downstream target of EDAR signaling (*Wnt10b*) during the periods coinciding with bud elongation (18 days post conception, dpc) and branching morphogenesis (postnatal day 5, P5). *Eda* mRNA is present in superficial urethral epithelium at 18 dpc and P5. *Edar* is expressed exclusively in prostate bud tips at 18 dpc, but found much more diffusely in the stroma at P5. *Wnt10b* is found only in prostatic bud distal tips at both 18 dpc and P5 (Fig. 1). Together, these results indicate that key EDAR signaling pathway components are present at the appropriate time and space to participate in prostatic ductal development.

**Fig. 2.**
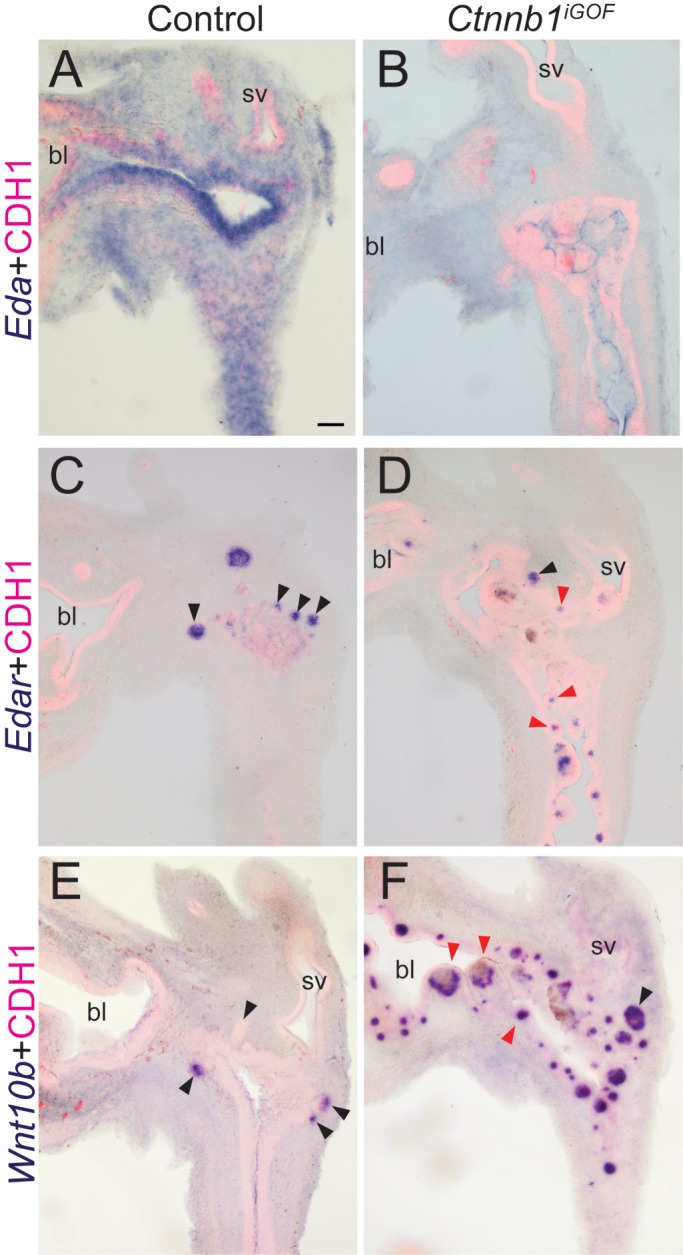
**CTNNB1 induces *Edar* and *Wnt10b* mRNAs in developing prostate.** Male *Shh^+/+^; Ctnnb1^tm1Mmt/tm1Mmt^* (control) and *Shh^creERT2/+^;Ctnnb1^tm1Mmt/tm1Mmt^* (*Ctnnb1^iGOF^*) embryos were exposed to tamoxifen and progesterone as described. Sections from three, 18 dpc male UGSs per genotype were stained by ISH to visualize (A,B) *Eda*, (C,D) *Edar*, (E,F) *Wnt10b* (purple). Sections were immunofluorescently counterstained to visualize epithelium marked by anti-cadherin 1 (CDH1) to facilitate tissue identification. bl, bladder; sv, seminal vesicle. Black arrowheads indicate prostatic buds. Red arrowheads indicate epithelial cell islands. Scale bar: 100 µm.

CTNNB1 drives expression of *Edar* and its downstream target *Wnt10b* (Zhang et al., 2009). To test whether CTNNB1 activates *Edar* and *Wnt10b* in developing prostate, we generated mice expressing activated the dominant stable *Ctnnb1^tm1Mmt^* gain-of-function (GOF) allele using *Shh^creERT2^* to generate *Ctnnb1^iGOF^* mice. The mice harbor a floxed *Ctnnb1* exon 3, which, when subjected to CRE*-*mediated recombination, encodes a functional and highly stable CTNNB1 protein form that accumulates in cells. *Cre* activity was activated by tamoxifen administration to the dam on 13 and 14 dpc. We showed previously that this tamoxifen dosing strategy does not in itself interfere with prostatic bud formation and that in this particular mouse strain, CTNNB1 accumulates in discrete cell islands readily discernable in tissue sections (Mehta et al., 2013). EDAR signaling pathway mRNAs were assessed at 18 dpc, after completion of prostatic budding in control mice. The *Edar* and *Wnt10b* mRNAs are noticeably more abundant in *Ctnnb1^iGOF^* mutant urethras compared to controls and localize to cell islands (Fig. 2) where we localized CTNNB1 overexpression (Mehta et al., 2013)*. Eda* mRNA is not detected within these cell islands and is noticeably less abundant in *Ctnnb1^iGOF^* mice compared to controls (Fig. 2). These results are consistent with CTNNB1 driving the expression of *Edar* and *Wnt10b* in the developing prostate.

**Fig. 3.**
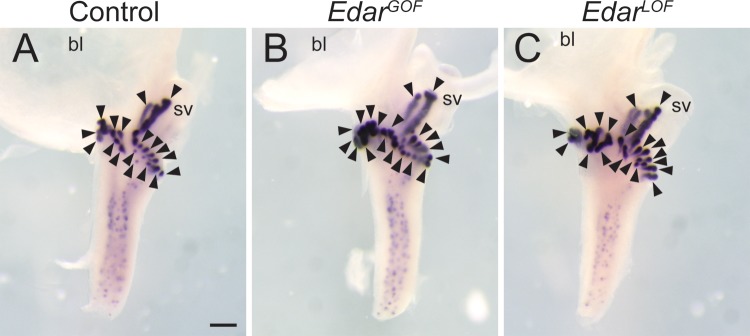
**EDAR is not required for prostatic bud formation and excessive EDAR does not change the number of prostatic buds formed.** Lower urinary tracts from P1 male (A) control, (B) *Edar* transgenic gain-of-function (*Edar^GOF^*), and (C) *Edar* transgenic loss-of-function (*Edar^LOF^*) mice were stained by ISH to visualize *Nkx3-1* (purple). Results are representative of three males per group. bl, bladder; sv, seminal vesicle. Arrowheads indicate prostatic buds. Scale bar: 500 µm.

CTNNB1 is required for prostatic bud formation (Mehta et al., 2013). To test whether *Edar* is required for the downstream activities of CTNNB1, we used a genetic approach involving *Edar* loss-of-function (LOF) mice that carry a missense mutation (Headon and Overbeek, 1999), and *Edar* transgenic GOF mice that overexpress *Edar* in the same cells where it is normally expressed (Mou et al., 2008). P1 male mouse lower urinary tracts were collected from each strain and stained in wholemount to visualize *Nkx3-1* marked prostatic buds. There are no appreciable strain-related differences in prostate bud size, organization, or number (Fig. 3). These results indicate that *Edar* is not required for prostatic bud formation, is not sufficient to induce changes in prostatic bud formation, and that CTNNB1 acts through an *Edar-*independent mechanism to control prostatic bud formation.

**Fig. 4.**
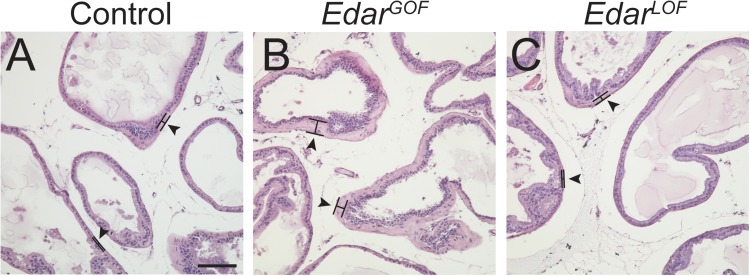
**Excessive EDAR causes increased periductal stromal thickness.** Mouse prostate tissue sections (5 µm) were generated from male control (A) or transgenic *Edar^GOF^* (B) or *Edar^LOF^* (C) mice. Sections were stained with Hematoxylin and Eosin and imaged using bright field microscopy. *Edar^GOF^* appear to have increased in the periductal, eosinophilic stromal layer. Arrowheads mark areas of altered periductal stroma thickness. Scale bar: 100 µm.

To identify potential roles for CTNNB1 and EDAR signaling in prostate maturation, we next examined adult prostates from control, *Edar^GOF^*, and *Edar^LOF^* mice. Prostates from these mice were examined at P50, after sexual maturation is complete. No gross abnormalities were observed in either mouse strain. All prostate lobes were present and appeared normal in location, size and morphology. Histological analysis revealed that prostate ducts from all groups are lined by a continuous luminal epithelial cell layer bounded by a basal epithelial cell layer (Fig. 4), evidence that prostate epithelial cell organization is grossly normal in *Edar^GOF^* and *Edar^LOF^* mice. However, *Edar^GOF^* mouse prostates exhibit an abnormally thickened and eosinophilic periductal stroma, indicative of high collagen content (Fig. 4).

**Fig. 5.**
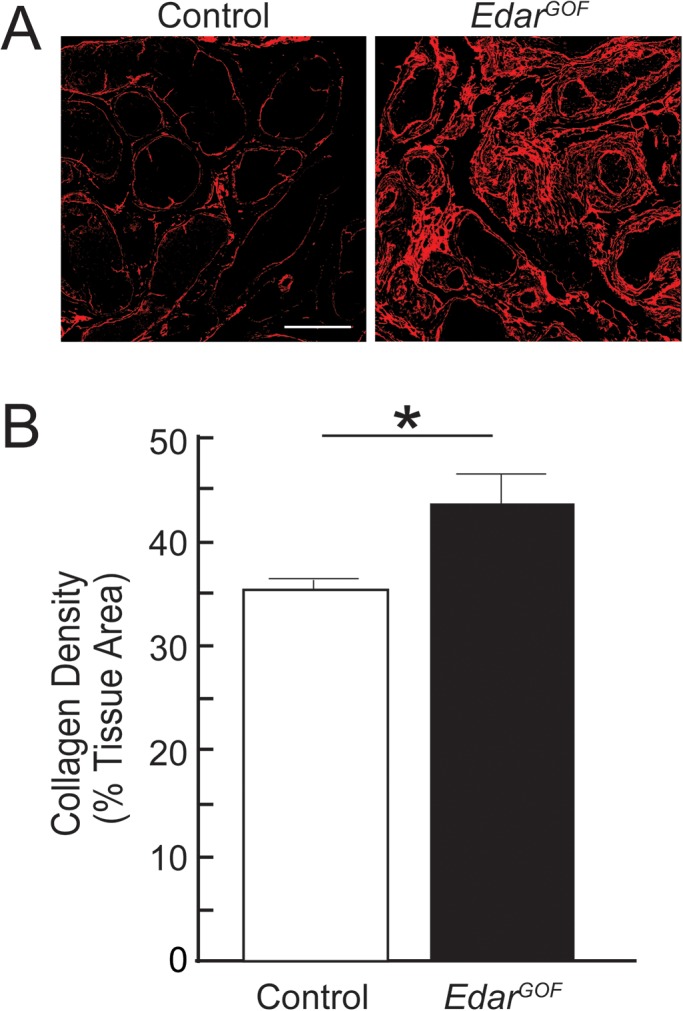
**Excessive EDAR increases dorsal prostate collagen density.** Mouse prostate tissue sections (5 µm) were generated from P50 male control or transgenic *Edar^GOF^* mice. (A) Sections were stained with Picrosirius Red and fluorescent imaging used to reveal collagen fibers and (B) quantify collagen density of both genotypes. Collagen density is the mean±s.e.m. of three non-serial sections from three litter-independent mice per group. Asterisks indicate significant differences from controls (Student's *t*-test, *P*<0.05). Scale bar: 100 µm.

We next evaluated collagen composition in adult male *Edar^GOF^* mutant mice. CTNNB1/Wnt signaling regulates extracellular matrix density in other organs including skin (Beyer et al., 2012; Lam and Gottardi, 2011; Wei et al., 2011), but how prostatic collagen is deposited and organized during prostate maturation has not been previously examined. Studies aimed at understanding prostate matrix regulation could reveal valuable insights into fibrotic prostatic disease, which has been associated with voiding dysfunction in adult men (Cantiello et al., 2013; Gharaee-Kermani et al., 2013; Ma et al., 2012). To visualize the prostatic collagen network, we used Picrosirius Red staining and fluorescent confocal microscopy in 5 μm tissue sections of *Edar^GOF^ and* paired wild-type controls of the same mouse strain. Automated fiber detection software (CT-FIRE) was used to measure individual collagen fiber metrics including density, orientation, alignment, diameter and length. Collagen architecture in *Edar^GOF^* prostates is significantly denser and more fibrous than in controls (Fig. 5).

## DISCUSSION

We found that CTNNB1 drives *Edar* expression in developing prostatic bud epithelium. CTNNB1 and EDAR were previously linked to ectodermal appendage development but this study links them in endodermal tissue development and sheds new light on our previous observation that CTNNB1-responsive target genes (*Lef1*, *Axin2* and *Wif1*) colocalize with *Edar* and *Wnt10b* mRNAs in elongating prostatic bud tips (Keil et al., 2012a, b; Mehta et al., 2011). It is not known whether EDAR is expressed in the terminal portion of buds from other endoderm-derived tissues, such as lung, or whether EDAR refines CTNNB1 signaling in these organs as it does in ectodermal appendages.

CTNNB1-dependent EDAR activation establishes size and periodicity of ectodermal appendages (Drew et al., 2007; Zhang et al., 2009). Though prostatic bud patterning also requires CTNNB1 (Francis et al., 2013; Mehta et al., 2013; Simons et al., 2012), we showed here that this process does not require EDAR. Mouse prostatic buds form in the normal quantity and size when *Edar* is genetically ablated and when *Edar* is overexpressed within its normal expression domain.

*Edar* LOF and GOF mutations do not appreciably affect prostate epithelial maturation but do impact prostatic stromal matrix organization in adult males. In control adult males, the interductal space is largely devoid of thick collagen bundles, with a majority of the collagen residing in the fibromuscular tunica around ducts, but adult *Edar^GOF^* mutant mouse prostates inappropriately accumulate collagen fibers. Given the timing of these changes, we conclude that EDAR is not necessary for early prostate formation but is needed for long term prostatic collagen homeostasis. How EDAR regulates prostatic collagens is still unclear. It is possible that EDAR signaling during prostatic development expands progenitors giving rise to collagen producing cells. Alternatively, indirect epithelial EDAR signaling during sexual maturation may drive stromal collagen deposition or reduce stromal collagen degradation. Prostatic matrix composition has drawn considerable clinical interest in recent years for its potential involvement in mediating lower urinary tract symptoms. Prostatic collagen density associates with lower urinary tract symptoms in aging men (Cantiello et al., 2013; Cantiello et al., 2014; Ma et al., 2012). While inflammatory mediators and metabolic syndrome are defined mediators of prostatic fibrosis (Gharaee-Kermani et al., 2012; Gharaee-Kermani et al., 2013; Wong et al., 2014), the role of other factors, including genetic factors, has not been examined. Our results provide the first evidence that excessive EDAR signaling during prostatic development can lead to an abnormally dense collagen matrix within the prostate and emphasizes the need to examine new pathways as potential therapeutic targets for prostate disease.

## MATERIALS AND METHODS

### Mice (*Mus musculus*)

All procedures were approved by the Animal Welfare and Ethical Review Body (AWERB) at the Roslin Institute, University of Edinburgh, in accordance with the United Kingdom Home Office Animal (Scientific Procedures) Act 1986, or the University of Wisconsin Animal Care and Use Committee. *Edar* transgenic gain-of-function (*Edar^GOF^*) mice were maintained on an FVB background and carry approximately 19 copies of a 200 kb yeast artificial chromosome (YAC) containing the entire mouse *Edar* gene, while homozygous *Edar^Tg951/Tg951^* (control) mice carry 36 copies (Mou et al., 2008). *Edar* mutant LOF (*Edar^LOF^*) mice were maintained on an FVB background and carry a G to A transition mutation causing a glutamate to lysine substitution in the death domain of the EDAR protein (E379 K) (Headon and Overbeek, 1999). Mice carrying the *Ctnnb1* exon 3 targeted deletion GOF allele (*Ctnnb1^tm1Mmt^*) were mated to wild-type mice (FVB/C57BL/6J mixed background) or to mice carrying *Shh^creERT2^* (*Shh^tm2(cre/ERT2)Cjt^*). To activate Shh*^creERT2^*, dams were injected with sterile corn oil (2.5 ml/kg *i.p.* maternal dose) containing 10% ethanol, tamoxifen (25 mg/kg maternal dose, Sigma-Aldrich #T56482) and progesterone (18.75 mg/kg maternal dose, Watson #NDC0591-3128-79) and dams were euthanized by CO_2_ asphyxiation. *Ctnnb1* gain-of-function (iGOF) *Ctnnb1^iGOF^* (*Shh^creERT2/+^*;*Ctnnb1^tm1Mmt/tm1Mmt^*) embryos were assessed together with their phenotypically normal paired littermate controls (*Shh^++^; Ctnnb1^tm1Mmt/tm1Mmt^*) (Brault et al., 2001; Harada et al., 1999; Harfe et al., 2004; Soriano, 1999). *Ctnnb1^tm1Mmt^* mice were from Dr Makoto Mark Taketo, Kyoto University. Wild-type FVB or C57BL/6J mice were acquired from The Jackson Laboratory. The morning of copulatory plug identification was considered E0.5. Genotyping was conducted as described previously (Mehta et al., 2013).

### Immunohistochemistry (IHC)

UGS tissues were fixed in 4% paraformaldehyde, dehydrated in alcohol, cleared in xylene, and infiltrated with paraffin as described previously (Mehta et al., 2011). 5 µm sections were generated and immunolabeled using an antibody against Cadherin 1 (1:200, Cell Signaling Technology; Cat# 3195, RRID: AB_10694492). Antibodies were validated in mouse urinary tract previously (Vezina, 2019). Immunolabeled tissues were mounted in anti-fade media (phosphate-buffered saline containing 80% glycerol and 0.2% *n*-propyl gallate) and imaged at 20× using a Nikon E600 microscope (Nikon; Tokyo, Japan).

### *In situ* hybridization

Detailed protocols are available at the GUDMAP database, www.gudmap.org and were described previously (Abler et al., 2011). Primer sequences for generating PCR-amplified probe templates are listed in Table S1. The staining pattern for each hybridized riboprobe was assessed in at least three litter-independent mice per genotype. Control and mutant tissues were processed together in the same tubes and as a single experimental unit to allow for qualitative comparisons among biological replicates and between genotypes or treatment groups.

### Hematoxylin and Eosin (H&E)

Adult prostate tissues were fixed in 4% paraformaldehyde, dehydrated in ethanol, cleared in xylene, infiltrated with paraffin and 5 µm sections were stained with Hematoxylin and Eosin. Brightfield imaging was performed using a Nikon E600 microscope (Nikon; Tokyo, Japan) using a 10× dry objective.

### Picrosirius Red (PSR)

Adult prostate tissues were fixed in 4% paraformaldehyde, dehydrated in alcohol, cleared in xylene, infiltrated with paraffin, and three non-serial 5 µm sections were taken from three litter-independent mice per group. Sections were taken from approximate equivalent tissue depth and stained with PSR as described previously (Wegner et al., 2017). Fluorescent imaging was performed using a SP8 Confocal Microscope (Leica; Wetzlar, Germany) using a 20× oil immersion objective (HC PL Apo CS2 NA=0.75; Leica). Samples were excited using a 561 nm laser and emission was detected between 635 and 685 nm. Laser intensity and gain were held constant between images. Images were captured at 1024×1024 resolution using LAS X software (Leica; Wetzlar, Germany). Tile scanning was used to stitch together images and generate an image across the entire prostate section. Total collagen density was determined by measuring the area of PSR stain in each section compared to the total cross-sectional area of the section. Automated fiber detection software (CT-FIRE) was used to measure individual collagen fiber metrics including density, orientation, alignment, diameter, and length. CT-FIRE is publicly available from the University of Wisconsin Laboratory for Optical and Computational Instrumentation.

### Statistics

For ISH experiments, experimental groups consisted of three to five UGSs from at least three independent litters. Images are representative of each treatment group. For PSR experiments, three non-serial dorsal prostate sections were imaged from three mice originating from three independent litters. A Levene's test was performed to test whether homogeneity of variances was the same between groups. A Student's *t*-test was conducted to identify differences between or among means using the Companion to Applied Regression (CAR) package for R (version 2.13.1) (Fox and Weisberg, 2011). Results are reported as mean±standard error of the mean (s.e.m.). For all statistical analysis, a difference of *P*<0.05 was considered significant.

## Supplementary Material

Supplementary information
