# Supplementary material for: Edar is a downstream target of beta-catenin and drives collagen accumulation in the mouse prostate
Source: Biol Open. 2019 Feb 11;8(3):bio037945. doi: 10.1242/bio.037945 (PMC6451354; doi:10.1242/bio.037945)
Supplement: Supplementary information [file biolopen-8-037945-s1.pdf]

Table S1. PCR primers used to synthesize digoxygenin-labeled riboprobes.

Click here to Download Table S1
